# The Associations Between Gaming Motivation and Internet Gaming Disorder: Systematic Review and Meta-analysis

**DOI:** 10.2196/23700

**Published:** 2022-02-17

**Authors:** Hsin-Yi Wang, Cecilia Cheng

**Affiliations:** 1 Department of Psychology The University of Hong Kong Hong Kong Hong Kong

**Keywords:** gaming motivation, problematic gaming, gaming disorder, video gaming, online gaming, compulsive gaming, escapism, culture, cross-cultural comparison, cultural individualism

## Abstract

**Background:**

There has been a surge in interest in examining internet gaming disorder (IGD) and its associations with gaming motivation. Three broad components of gaming motivation have been proposed: achievement, immersion, and social. Achievement-oriented players are motivated by gaining in-game rewards, immersion-oriented players are motivated by the experience of immersion in the virtual world, and social-oriented players are motivated by the need to socialize with other players through gaming.

**Objective:**

This study aimed to (1) quantitatively synthesize the growing body of literature to systematically examine the discrepancies in the magnitude of associations between various components of gaming motivation and IGD and (2) examine the moderating role of cultural dimension on the association between escapism gaming motivation and IGD.

**Methods:**

We conducted a systematic search of multiple databases between 2002 and 2020. Studies were included if they (1) included quantitative data, (2) used measures assessing both gaming motivation and IGD, and (3) contained sufficient information for effect size calculation.

**Results:**

The findings revealed IGD to have a stronger association with achievement motivation (*r*=0.32) than with immersion (*r*=0.22) or social motivation (*r*=0.20), but the strongest such association was found to be with escapism motivation (*r*=0.40), a subcomponent of immersion motivation. Our cross-cultural comparison further showed a stronger association between escapism motivation and IGD in studies conducted in individualistic (vs collectivistic) regions.

**Conclusions:**

This meta-analysis highlights the importance of acknowledging the discrepancies among different components of gaming motivation with respect to their role in the development of IGD, as well as the potential cultural variations in the strength of such associations.

## Introduction

### Background

Internet gaming is a popular leisure activity in the present digital age. However, gaming can become problematic when it interferes with a player’s psychological and social functioning [1,2]. Considering the prevalence of this emergent problem worldwide [3,4], problematic gaming was included as a “condition for further study” in the Diagnostic and Statistical Manual of Mental Disorders, Fifth Edition (DSM-5), under the label of “*Internet Gaming Disorder”* (IGD) [5], and considerable research efforts have been made to conceptualize and assess IGD [6,7]. After IGD was listed as a “condition for further study” in the DSM-5 in 2013, *gaming disorder* was also officially included in the International Classification of Diseases, Eleventh Revision (ICD-11) in 2018. Thus far, only 1 measure has been developed based on the ICD-11 criteria, but none of the studies retrieved in this meta-analysis used this measure. This work thus focuses on IGD because several measures included in the present meta-analysis were constructed based on the DSM criteria.

Some scholars conceptualize this disorder to be a form of behavioral addiction because some of its symptoms are similar to those of other behavioral addictions (eg, gambling disorder) and substance use disorder [8]. Based on the criteria for other behavioral addictions, several recurring symptoms of IGD have been identified, including preoccupation with gaming activities and withdrawal symptoms such as irritability when individuals are kept from playing [9,10]. Studies have revealed that IGD was positively associated with mental health issues such as depression, social anxiety, and psychological distress [11-16].

### Gaming Motivation as an Underlying Factor of IGD

As gaming is generally regarded as a healthy leisure activity, scholars have advocated for an examination of IGD from the viewpoint of etiological factors such as gaming motivation [17,18]. Although the specific gaming motivation differs considerably among players, the driving force behind IGD stems from players’ over-reliance on gaming to fulfill certain psychological needs [19], a postulation corroborated by evidence on the positive associations between various types of gaming motivation and IGD [20,21]. Nevertheless, several issues remain unexplored, and we conducted a meta-analysis to quantitatively synthesize the available evidence to address 2 of these important but unresolved issues.

The first issue is the empirical inconsistencies regarding the associations between gaming motivations and IGD. For instance, some studies indicate that certain types of gaming motivation, such as the desire to achieve in-game success, are more strongly associated with IGD than are other types of gaming motivation, such as the motivation to form social relations through gaming [22,23]. However, the evidence remains inconclusive because many studies in this area have examined only a single type of gaming motivation, thereby rendering between-motivation comparisons impossible [20]. Second, cross-cultural comparisons are essential for studying IGD as a global phenomenon [24]. For instance, although intervention programs for IGD are more commonly observed in Asian countries characterized by collectivistic orientations [25-27], the implications and the potential influence of such cultural differences on the association between gaming motivation and IGD has yet to be empirically investigated.

### Components of Gaming Motivation

To identify specific components of gaming motivation that play an influential role on IGD, we adopted Yee’s categorization [28] as our theoretical foundation for a systematic analysis. Among the various frameworks available in the literature, we chose Yee’s framework [28] because it is the most comprehensive one that adopts a hierarchical structure encompassing both components and subcomponents and is also by far the most widely adopted framework in the literature of gaming motivation. This tridimensional framework categorizes gaming motivation into 3 broad components. First, achievement motivation comprises gaming motivations pertaining to the desire to achieve in-game recognition, power, and status. Second, social motivation refers to gaming motivations related to the urge to build connections and interact with other players. Third, immersion motivation includes those motivations pertaining to the desire to experience the virtual in-game world.

The hypothesized discrepancies among the achievement, social, and immersion motivation components are corroborated by prior studies indicating that the 3 components have distinct magnitudes of associations with IGD. For instance, the achievement component has been consistently identified as a robust indicator of IGD, with a moderately positive association between the 2 constructs [22,29]. Specifically, scholars have postulated that players who lack success in real life often choose to compensate for such deficiencies with their achievements in the gaming world [30]. Hence, players who overvalue their in-game achievements often need to endure extensive gameplay to achieve the occasional moments of success, which in turn increases their propensity to develop IGD [30].

Another body of studies has revealed weak to moderate positive associations between immersion motivation and IGD [23,31]. Immersion motivation entails the incentive to immerse oneself in gaming, as the highly immersive nature of internet games may serve as a refuge for players to escape from real-life difficulties (eg, social anxiety, loneliness) [32,33]. Although gaming may provide temporary relief from such difficulties, it can also create problematic beliefs about the maladaptive use of gaming to constantly avoid real-life issues [19].

Mixed findings, however, have been reported for the association between social motivation and IGD. Although some studies have documented weak to moderate positive associations between the 2 constructs [29,34], others have reported null or even contradictory findings [22,35]. Such inconsistencies may be related to the complex social outcomes of gaming. For instance, although players with a stronger social motivation are more likely to receive online social support through gaming [36,37], these online relations have also been linked to the social “obligation” of gaming [38]. More specifically, players who want to become core members of a gaming team are often obligated to play for as long as other team members want to play. This perceived obligation of gaming often increases players’ gameplay time and gaming-related distress, both of which contribute to the onset of IGD [30,39].

Studies examining all 3 motivation components have compared the associations of these components with IGD. For example, studies investigating both achievement and immersion motivations have revealed IGD to be more strongly associated with the former than the latter [22,29]. Moreover, IGD has also been consistently shown to have a weaker association with social motivation than with achievement motivation [40] or immersion motivation [23].

Based on these empirical findings, we hypothesize that the 3 components of gaming motivation are differentially associated with IGD. More specifically, we predict that this meta-analysis may reveal the association between achievement motivation and IGD to be stronger than that between immersion motivation and IGD. Moreover, social motivation is expected to have a weaker association than achievement or immersion motivation with IGD.

### Subcomponents of Gaming Motivation

In addition to the 3 motivation components conceptualized in the tridimensional gaming motivation framework, each motivation component also comprises multiple subcomponents. Moreover, prior studies have revealed sizable differences among specific subcomponents of the same motivation component with respect to their association with IGD [41,42]. To empirically evaluate these within-component variations, we adopted Yee’s 10-factor motivation taxonomy [43] that includes 10 motivation subcomponents, each of which is classified under 1 of the 3 aforementioned gaming motivation components (ie, achievement, immersion, and social).

According to the 10-factor taxonomy, immersion gaming motivation comprises 4 subcomponents: discovery, role-play, customization, and escapism. Discovery motivation refers to players’ interest in exploring the virtual in-game world, whereas role-play motivation reflects players’ incentive to create and identify with their in-game avatars. Customization motivation refers to players’ desire to alter various aspects of their in-game characters as they wish, whereas escapism motivation reflects their urge to use gaming activities to escape from real-life difficulties. Studies have revealed considerable differences in the association of each of the 4 subcomponents with IGD. For example, escapism motivation has been consistently documented to have a moderate to strong association with IGD [43], whereas the discovery and customization motivation subcomponents display a much weaker such association [44,45].

These within-component variations have been attributed to the stronger conceptual connections between escapism motivation and IGD than between the other subcomponents and IGD [32]. According to Baumeister’s escape-from-self theory [46], the assertion that immersion-oriented players use gaming to evade real-life adversities is more applicable to escapism motivation [47], primarily because that subcomponent refers explicitly to such desire [48]. In contrast, the other subcomponents, such as discovery and customization motivations, do not entail similar incentives to use gaming as a means to escape. It is thus important to disentangle the distinctions between escapism motivation and the other subcomponents of immersion motivation in this meta-analysis.

In contrast to the substantial differences among the subcomponents of immersion motivation, there are fewer distinctions among the subcomponents of achievement motivation and social motivation with respect to their association with IGD. There are 3 subcomponents of achievement motivation. Advancement motivation refers to the desire to accumulate in-game rewards, mechanics motivation describes the urge to understand the gameplay mechanism, and competition motivation refers to the incentive to challenge and outcompete other players. Studies have shown moderately positive associations between all 3 subcomponents and IGD [42,49].

Social motivation also comprises 3 subcomponents. Socializing motivation describes players’ intention to establish casual social relations with other players, relationship motivation refers to players’ desire to maintain long-term social relations through gaming, and teamwork motivation describes players’ interest in collaborating with other players during gameplay. Past studies have revealed few differences among the 3 social motivation subcomponents with respect to their associations with IGD [50,51].

Considering these findings, we hypothesize that there will be significant differences among the immersion motivation subcomponents in terms of their associations with IGD, but there will be no such significant differences among the subcomponents of achievement motivation or those of social motivation.

### Cultural Dimensions

We further posited that the magnitude of the association between gaming motivation and IGD varied by some cultural factors. Cross-cultural psychologists have maintained that people’s thoughts and behaviors are often shaped by the culture in which they reside [52,53]. Specifically, one of the most widely studied cultural dimensions is individualism-collectivism [54,55]. In individualistic cultures, the self-interest of individuals is generally regarded to be of greater importance than the collective interests of the group, whereas in collectivistic cultures, it is the collective interests of the group that take precedence [56,57].

We hypothesize that the cultural dimension of individualism-collectivism may also explain the differential magnitudes of the association between IGD and gaming motivation, escapism motivation in particular. Such assertion is made based on past studies that documented cross-cultural differences in the way that members of a society interpret the non-normative behaviors such as excessive gaming. For instance, members of individualistic societies tend to view behavioral addiction or substance addiction as a consequence of one’s own choices or personality, whereas members of collectivistic societies tend to attribute it to such interpersonal factors as the influence of the social environment or such institutional factors as government policy [58].

In the context of IGD, such cultural differences are reflected in the preventive approaches implemented in different regions. For example, legislation concerning prevention programs for IGD is more commonly observed in collectivistic countries such as Japan and South Korea [59]. These regions place stronger emphasis on the need for parental and teacher supervision of youngsters’ gaming activities [60,61]. Such policies are less common in Western individualistic countries. Hence, in societies emphasizing collectivistic values, players with stronger escapism motivation may more likely receive attention from their parents or teachers and benefit from prevention programs before their gaming activities evolve into problematic or addictive gaming. In individualistic societies, where similar prevention programs are less accessible, players who wish to escape real-life issues through gaming may not receive sufficient social support and attention to prevent the onset of IGD. In summary, we hypothesize that the association between escapism motivation and IGD may be stronger in studies conducted in individualistic (vs collectivistic) regions.

## Methods

### Literature Search

The PRISMA (Preferred Reporting Items for Systematic Reviews and Meta-Analyses) guidelines were adhered to when this meta-analysis was performed and when the findings were reported. To identify relevant reports for this meta-analysis, we performed a multistage literature search using diverse methods in April 2019 with an update in March 2020. In the first stage, electronic bibliographic searches were conducted using 3 meta-databases: ProQuest, EBSCOhost, and Scopus. The following Boolean string was used in the search for relevant articles: (“Internet gam*” OR “online gam*” OR “digital gam*”) AND (“disorder” OR “abuse” OR “addict*” OR “compulsi*” OR “dependenc*” OR “excess*” OR “pathologic*” OR “problematic”).

In the next stage, we manually located reports that could not be identified in the first stage. First, the reference sections of previous meta-analyses related to the topic of IGD were scanned [3,6]. Second, bibliographic searches were performed on the aforementioned meta-databases for studies citing instruments of IGD and gaming motivation. Third, a review of the “gray literature” was performed to identify relevant unpublished reports (eg, regional literature databases, online archives). In the final stage, we contacted authors of some reports identified for further clarification and the provision of missing data.

### Study Inclusion and Exclusion Criteria

To be included in this review, reports identified in the literature search had to contain at least one quantitative measure of IGD and at least one quantitative measure of gaming motivation. Reports were excluded if they (1) included qualitative data alone, (2) used measures assessing only the problematic use of internet activities or offline gaming but not internet gaming, (3) employed a measure of either IGD or gaming motivation but not both, or (4) lacked sufficient information for effect size calculation. No exclusions were made based on language.

### Report Selection Process

**Figure 1:**
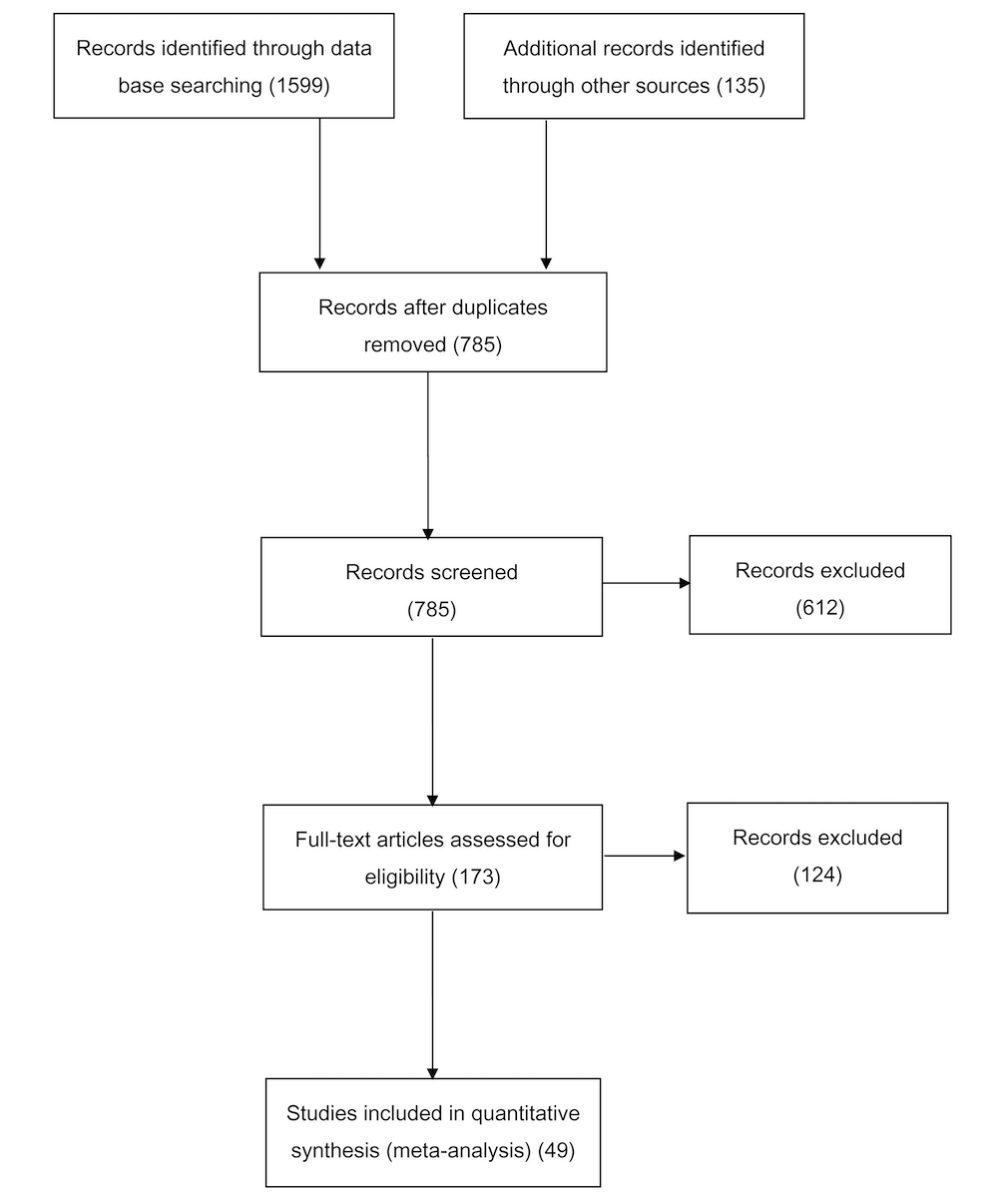
PRISMA (Preferred Reporting Items for Systematic Reviews and Meta-Analyses) diagram illustrating the procedures of report selection in the meta-analysis.

Two independent reviewers performed the initial screening of the reports based on their titles, abstracts, keywords, or a combination thereof. After the initial screening, the full text of the remaining reports was retrieved for data extraction. Figure 1 presents a PRISMA flow diagram illustrating the report selection process, which yielded the final pool of 49 eligible reports.

### Coding of Effect Sizes and Moderators

#### Data Extraction Process

The 2 reviewers who performed the screening task also independently extracted the following data: number of participants, sex and age composition, research design, geographical location of the study, measures of IGD and gaming motivation used, gaming motivation components and subcomponents, and the corresponding effect size estimates for the associations between IGD and gaming motivation. Any discrepancies in coding were resolved by a discussion with the senior author who was not involved in the coding process. The interrater reliability index of Krippendorff α coefficient was .92 [62], indicating a high level of interreviewer reliability.

#### Effect Size Metric

The Pearson product-moment correlation coefficient (*r*) was selected as the effect size metric for its ease of interpretation. When such an effect size could not be found, other relevant statistics (ie, chi-square, Cohen *d*) were extracted and then converted into *r* using the “compute.es” statistical package in the R programing environment (version 4.0.0; R Foundation for Statistical Computing) [63,64].

#### Coding of Effect Size Estimates

The reviewers extracted all relevant effect size estimates pertaining to the associations between gaming motivation and IGD. We extracted all effect size estimates of the associations of IGD with the 3 gaming motivation components (ie, achievement, immersion, and social) and IGD and with the gaming motivation subcomponents and IGD based on the aforementioned 10-factor motivation model [28]. For the studies that used instruments based on this model or other conceptually similar models, effect size estimates were extracted.

### A Priori Moderators

All the a priori moderators described in the Introduction were coded during data extraction. First, the motivation component was coded as a 3-level categorical variable (ie, achievement, immersion, and social). Second, the subcomponents categorized under each motivation component were coded as a categorical variable. For instance, the subcomponents related to achievement motivation were coded as advancement, mechanics, and competition, and those related to social motivation were coded as socializing, relationship, and teamwork. Those related to immersion motivation were coded as a 4-level categorical variable: discovery motivation, role-playing motivation, customization motivation, and escapism motivation.

Finally, the moderator variable of individualism was defined as a continuous variable, with higher scores assigned to regions with higher individualistic cultural values. We first recorded the geographical locations in which individual samples were recruited and then ascribed a national individualism score to each sample based on Hofstede’s data matrix [65].

### Meta-analytic Procedures

Three-level mixed-effects meta-analysis was adopted in this study. Conventional meta-analytic methods may generate biased effect size estimates when effect sizes are not independent. In this meta-analysis, there were many reports (77%) with multiple effect sizes for the associations between the various components of gaming motivation and IGD. It was thus necessary to perform 3-level meta-analysis using the structure of a multilevel model, with level 1 analyzing effect sizes, level 2 analyzing independent samples, and level 3 analyzing individual reports [66].

Main effect analysis was conducted using the metaSEM package version 1.2.0 [67] and metafor package version 2.4.0 [68] in the R programming environment [64]. Such analyses examined the magnitude and direction of the association of IGD with the various gaming motivation components and subcomponents. Maximum likelihood estimation was performed to estimate 95% CI.

The Cochran *Q* statistic was examined to evaluate the presence of heterogeneity and determine whether the effect size was consistent across reports. In addition, the *I*^2^ index was also calculated to evaluate between-study heterogeneity. If both the *Q* statistic and *I*^2^ index indicated a significant level of heterogeneity, 3-level mixed-effects meta-analysis was performed to further examine the variability across reports using the metaSEM package.

### Detection of Possible Bias Risks (Omnibus Analysis)

#### Study Quality

The quality of each report was evaluated based on 8 indicators [69,70]: sampling method, sample heterogeneity, statistical power, sample description, measurement validity, measurement reliability, study methodology, and study design. The criteria used for coding the various indicators are summarized in Table 1. An individual report was assigned a value of 1 for meeting the criteria of each indicator and a value of 0 for failing to do so, except for scale reliability and scale validity, both of which were coded as continuous variables. To determine whether the quality of individual reports exerted an influence on the magnitude of a correlation, we tested the moderating effects of study quality using the 8 individual indicators and the composite score.

**Table 1:** Coding criteria for study quality indicators.

Quality indicators^a^	Criteria met (score=1)	Criteria not met (score=0)
Sampling method	Sampling involved random selection of participations	Sampling involved nonrandom methods
Sample heterogeneity	Sample comprised 2 or more demographic groups	Sample included only a single group
Statistical power	Sample size was large enough to yield adequate statistical power	Sample size was not sufficient to yield adequate statistic power
Sample description	Demographic information was clearly described	Demographic information was not clearly described
Measurement validity	All scales used in the study were validated	None were validated
Measurement reliability	All scales were reliable (Cronbach α≥.70)	No scales were reliable (Cronbach α≥.70)
Study methodology	Study adopted 2 or more methodologies	Study adopted only 1 methodology
Study design	Data were collected at 2 or more time points	Data were collected at only 1 time point

^a^The indicators of scale reliability and scale validity were coded as continuous variables (ie, percentage of scales that were reliable or valid, respectively). The other indicators were all coded as dichotomous variables.

#### Publication Bias

To address the potential issues of publication bias, we adopted several common methods. First, the fail-safe *N* was employed to estimate the number of unpublished or missing reports with an effect size averaging 0 that would nullify the effect observed in the meta-analysis. Publication bias was a potential concern if the fail-safe *N* was smaller than 5*k* + 10 (*k* = the number of studies) based on established criteria [71]. Second, publication bias was evaluated by funnel plots and Egger tests derived from weighted regression [72], with asymmetrical funnel plots indicating publication bias. The potential issue of asymmetry was also indicated by significant Egger test results. Finally, we employed the trim-and-fill technique, which estimates the number of missing reports and provides an estimate of the effect size after publication bias has been taken into account [73]. If the adjusted effect size is vastly different from that yielded prior to such adjustment, publication bias is a likely concern. These analyses were performed using the metafor package in the R programming environment.

## Results

### Descriptions of the Data Set

The majority of the 49 studies included in this analysis were published in academic journals (89%), and the remainder included 6 unpublished dissertations and 2 conference proceedings. Most were published after 2008, when studies on IGD began to be seen. Moreover, 74% were published after 2014, when the American Psychiatric Association listed IGD in the DSM-5 as a condition for further study [74].

The aggregate number of participants in the meta-analysis of this study was 52,254 (mean 1022, *SD* 1249.37, range 59-5222). The participants were recruited from 86 independent samples. The mean age across the samples was 26.51 (*SD* 5.03, range 12.90-34.23), and 68% of the participants were men. The samples were recruited from 23 countries across multiple geographical regions, including Europe (54%), Asia (25%), North America (16%), and other regions (5%).

To assess IGD symptoms, some of the included studies adopted the DSM criteria [74]. Other popular such measures included the Addiction-Engagement Questionnaire [75], Game Addiction Scale [76], Internet Gaming Disorder Test [7], and Problematic Online Gaming Questionnaire [77]. Moreover, some studies adapted Young’s Internet Addition Test [78] to measure IGD symptoms. To assess gaming motivation, most of the included studies employed the Motivation to Play in Online Games Questionnaire [28], followed by the Motives for Online Gaming Questionnaire [79] and Online Gaming Motivations Scale [80]. All the subscales of these gaming motivation measures can be mapped onto Yee’s hierarchical model [28], except for the “fantasy” subscale. The results involving this subscale were omitted in this meta-analysis.

### Main-Effect Meta-analysis

#### Gaming Motivation Components and IGD

To examine the associations between the 3 gaming motivation components (ie, achievement, social, and immersion) and IGD, 3 sets of 3-level meta-analysis were performed based on 64 effect size estimates extracted from 22 reports. The results are summarized in Table 2. Significant positive correlations were identified for the association between each gaming motivation component and IGD. The correlation between the achievement motivation and IGD was 0.32, indicating a medium effect size, whereas that between the other 2 types of motivation and IGD was small to medium in magnitude (*r*=0.20 and *r=0*.22).

As shown in Table 2, the Cochran’s *Q* test results revealed significant heterogeneity in the effect size estimates of all 3 gaming motivation components. In addition, the *I*^2^ statistics indicated a moderate to high degree of between-study heterogeneity [81]. Accordingly, 3-level mixed-effects meta-analysis was performed to examine the hypothesized moderating effects of the 4 a priori moderators.

**Table 2:** Correlation coefficients of problematic gaming with gaming motivation components and subcomponents. Subscripts that do not share a common number (for comparisons among components) or symbol (for comparisons among subcomponents) differ significantly at *P*<.05.

Gaming motivation	*k*^a^ value	*r*^b^ value	95% CI value	*Q*^c^ value	*I*^2d^ value	*I*^2e^ value
Achievement component^f^	21	0.32^g^_1_	0.27 to 0.36	182.21^g^	0.91	0.03
Advancement subcomponent	10	0.29^g^_*_	0.25 to 0.35	310.14^g^	0.52	N/A^h^
Mechanics subcomponent	9	0.28^g^_*_	0.25 to 0.33	11.01	0.36	N/A
Competition subcomponent	24	0.31^g^_*_	0.23 to 0.42	1341.25^g^	0.74	0.11
Social component^i^	27	0.20^g^_2_	0.14 to 0.29	844.90^g^	0.78	0.18
Socializing subcomponent	27	0.17^g^_#_	0.09 to 0.26	1291.76^g^	0.84	0.12
Relationship subcomponent	11	0.25^g^_*_	0.13 to 0.32	101.42^g^	0.88	N/A
Teamwork subcomponent	8	0.08_$_	−0.09 to 0.22	145.01^g^	0.49	N/A
Immersion component^j^	16	0.22^g^_2_	0.13 to 0.30	341.22^g^	0.80	0.13
Discovery subcomponent	8	0.07_$_	−0.01 to 0.17	40.87^g^	0.41	N/A
Role-play subcomponent	24	0.22^g^_#_	0.14 to 0.32	2143.22^g^	0.54	N/A
Customization subcomponent	8	0.21^g^_#_	0.15 to 0.30	50.41^g^	0.41	N/A
Escapism subcomponent	44	0.40^g^_*_	0.35 to 0.45	1510.22^g^	0.70	0.21

^a^*k:* number of tested correlations.

^b^*r:* pooled correlation coefficient.

^c^*Q:* Cochrane heterogeneity statistic.

^d^*I*^2^: level 2 heterogeneity index.

^e^*I*^2^: level 3 heterogeneity index.

^f^Comprises the advancement, mechanics, and competition subcomponents.

^g^*P*<.001.

^h^N/A: not applicable.

^i^Comprises the socializing, relationship, and teamwork subcomponents.

^j^Comprises the discovery, role-play, customization, and escapism subcomponents.

#### Gaming Motivation Subcomponents and IGD

As summarized in Table 2, significant positive correlations were identified for 8 of the subcomponents (*r* ranging from 0.17 to 0.40), but those for the teamwork and the discovery subcomponents were not significant (*r*=0.08 and *r*=0.07, respectively).

The magnitude of the correlations between all 3 subcomponents of achievement motivation and IGD was moderate (*r*= 0.29 for advancement, *r*=0.28 for mechanics, and *r*=0.31 for competition). For the subcomponents of social motivation, the magnitude of the correlations with IGD was small to moderate for socializing and relationship motivation (*r*=0.17 and *r*=0.25, respectively) but not significant for teamwork motivation (*r*=0.08). The results for the subcomponents of immersion motivation were more diverse. The association between escapism motivation and IGD showed the strongest correlation (*r*=0.40), whereas the magnitude of the correlations with IGD was small to moderate for the role-play and the customization subcomponents (*r*=0.22 and *r*=0.21, respectively). The discovery motivation subcomponent was found to have no significant association with IGD (*r*=0.07).

The Cochran *Q* heterogeneity statistics indicated significant between-study heterogeneity for all the gaming motivation subcomponents except mechanics motivation (*Q*=20.14, *P*=.08). Similarly, the *I*^2^ statistics also revealed a moderate to high degree of between-study heterogeneity in the effect size estimates [81].

### Moderator Analysis

#### Moderating Role of Gaming Motivation Components

For the moderator of the gaming motivation component, the findings from the 3-level mixed-effects meta-analysis supported our hypothesis that this variable moderated the association between gaming motivation and IGD (*Q*_M_=1199.27, *P*<.001). Pairwise comparisons indicated IGD to have a significantly stronger correlation with achievement motivation than with social or immersion motivation (all *P*<.001). However, only a marginally significant difference between the social motivation–IGD correlation and the immersion motivation–IGD correlation was identified (*P*=.15).

#### Moderating Role of Gaming Motivation Subcomponents

A series of 3-level mixed-effects meta-analysis was performed to examine the moderating effects of the gaming motivation subcomponents of the 3 motivation components.

In the first set, the results corroborated our hypothesis that the subcomponents of achievement motivation were not a significant moderator (*Q*_M_=9.81, *P*=.09). In the second set, however, the results were contrary to our hypothesis concerning the subcomponents of social motivation, indicating that they significantly moderated the association of social motivation with IGD (*Q*_M_=45.94, *P*=.01). Pairwise comparisons indicated significantly stronger correlations of IGD with socializing motivation than with relationship or teamwork motivation (all *P*<.01), and significant differences were identified between the relationship motivation–IGD correlation and the teamwork motivation–IGD correlation (*P*<.001).

In the third set, the results supported the hypothesized moderating effect of the subcomponents of immersion motivation (*Q*_M_=602.84, *P*<.001). Further analysis based on pairwise comparisons revealed the correlation of IGD with escapism motivation to be significantly stronger than that with discovery, role-play, or customization motivations (all *P*<.001). Although no significant differences were identified between the role-play motivation–IGD correlation and the customization motivation–IGD correlation (*P*=.23), the correlation of IGD with the role-play and customization motivation subcomponents was significantly stronger than that with the discovery motivation subcomponent (*P*<.001).

#### Moderating Role of Individualism (vs Collectivism)

Three-level mixed-effects meta-analysis was also conducted to examine the hypothesized moderating role of individualism. The results supported our hypothesis that the cultural dimension of individualism had a significant moderating effect (*b*=−.0023, *SE* .0015; *P*<.001) on the correlation between the escapism subcomponent of immersion motivation and IGD. Simple slope analysis further revealed the positive correlation between IGD and escapism motivation to be stronger among the samples recruited from regions higher versus lower in individualism (*r*=0.47 vs *r*=0.25).

### Detection of Possible Bias

#### Study Quality

Moderator analysis was performed to examine the potential influence of study quality on the meta-analysis results based on 8 indicators. The nonsignificant results revealed that study quality did not exert a significant influence on the association between any of the components or subcomponents of gaming motivation and IGD (all *P* ranging from .16 to .47).

#### Publication Bias

The various analyses similarly revealed no evidence of publication bias. First, the fail-safe *N* estimations computed based on the revisited file-drawer test (*N* ranging from 583 to 12,770) all exceeded the suggested cutoff value (5*k* + 10 = 255). Moreover, the trim-and-fill technique showed no evidence to suggest that publication bias would substantially alter the effect size estimates after the corresponding adjustments. Finally, the Egger regression test revealed no significant asymmetry (all *P* ranging from .09 to .68), indicating that the effect size estimates were unrelated to sample size.

## Discussion

### Summary of Findings

This meta-analysis synthesized the growing body of literature and addressed several unresolved research issues in the study of gaming motivation and IGD. First, the findings partially support the hypothesized discrepancies among the 3 gaming motivation components (ie, achievement, social, and immersion) with regard to their associations with IGD. Specifically, IGD tends to have a stronger association with achievement motivation than with immersion motivation or social motivation. However, there is no significant difference between immersion motivation and social motivation, with both showing a weak to moderate positive correlation with IGD. Hence, social motivation should also be acknowledged in the treatment of this emergent disorder.

Regarding the within-component variations, there are no significant differences among the subcomponents of achievement with regard to their associations with IGD. The results for the immersion motivation component are consistent with the escape-from-self theory [46], with the escapism motivation subcomponent demonstrating a considerably stronger association than the other subcomponents (ie, discovery, customization, and role-play motivations) with IGD. Moreover, interest in experiencing the virtual world (ie, discovery motivation) is not identified as a risk factor for IGD. This meta-analysis thus highlights the importance of distinguishing between motivation to explore the virtual world and motivation to use gaming to escape from various real-life issues.

The results for the social motivation component contradict our hypothesis. Our findings show that the relationship subcomponent has a considerably stronger association than the socializing or the teamwork subcomponent with IGD. These findings substantiate the postulation that “social obligation” can contribute to excessive gameplay in socially oriented players [38]. Although the collaborative gameplay design of many online games may increase players’ online social capital [37,82], it can require players to unwillingly engage in gaming for the collective benefit of the gaming team [83]. Such obligation can lead to excessive gameplay and decrease the resources needed to maintain offline social relations [84,85]. Moreover, the nonsignificant association between the teamwork subcomponent and IGD further indicates that collaborative gameplay itself does not directly influence the onset of IGD. Thus, only the socializing and the relationship motivation subcomponents should be acknowledged as relevant to IGD and its treatment.

### Implications for Assessment and Treatment

This meta-analysis has implications for researchers. Specifically, the considerable discrepancies identified among the subcomponents of social motivation and immersion motivation highlight the importance of assessing gaming motivation at the subcomponent level. The 3 broad components of gaming describe 3 distinct domains of motivation for gameplay, but these domains also encompass multiple subcomponents that need to be evaluated in diverse ways. For instance, exclusive examination of the social motivation component may lead to neglect of the differences between players’ interest in forming social relations and their interest in collaborating with other players. To provide a more comprehensive examination of gaming motivation, future studies should investigate gaming motivation at both the component and subcomponent levels.

Our meta-analysis also has implications for clinicians and practitioners. Some scholars have maintained that a shortcoming of current cognitive therapy–based interventions is the lack of measures to evaluate the cognitions associated with IGD [86,87]. To bridge this knowledge gap, discerning among the multiple motivations for gaming may help practitioners identify the underlying problematic cognition and use specific treatment programs. For example, clients with a stronger social gaming motivation may more likely believe gaming to be the only means of forming social relations; thus, it may be helpful to incorporate programs that strengthen their social skills in face-to-face interactions [27,88]. However, reviews of IGD treatment programs indicate that gaming motivation is rarely considered [86]. Thus, practitioners should consider evaluating their clients’ gaming motivation and then tailor treatment programs to specifically address the corresponding problems.

#### Research Caveats and Directions for Future Research

As our findings are limited by the available studies, there are several research caveats. First, it is noteworthy that Yee’s motivation taxonomy [28] was originally developed from samples of massively multiplayer online role-playing game players, so some of its motives (eg, role-play, customization) are specifically related to this game genre. Some studies have revealed certain genres (eg, massively multiplayer online role-playing game, first-person shooter) to have a stronger tendency to elicit IGD among players with specific types of gaming motivation, including social and immersion motivation [21]. In response to the increasingly diversified gameplay designs, some scholars have called for greater effort in investigating the role of game genre in the motivation and behavior across players of various game genres [89,90]. Moreover, although most subscales of the included gaming motivation measures are covered in Yee’s framework [28], there are a few exceptions (eg, fantasy) that cannot be mapped onto the framework. Researchers may integrate Yee’s framework with other existing ones to broaden coverage.

Second, a large number of studies included in our meta-analysis recruited players who are highly committed to gaming, so our findings are not necessarily generalizable to the increasing number of casual players who play occasionally, as these players may have distinct interpretations of gaming motivations [91]. For example, committed players tend to perceive social gaming motivation as their desire to build and maintain online social relations through gaming activities [92]. For casual players, however, this type of gaming motivation primarily indicates their interest in playing with members of their offline social circle in a group context [91]. Thus, the scope of future studies should be expanded to include different types of players (eg, committed vs casual players) to allow comparisons of their potentially distinct interpretations of gaming motivation.

Third, as our meta-analysis mainly included studies that examined IGD based on composite scores for multiple symptoms, our findings may not be generalizable to specific symptoms of IGD [24,93] such as preoccupation with gaming activities or unsuccessful attempts to control gaming activities. Studies using composite scores for multiple symptoms may be unable to distinguish among the varying magnitudes of the association of gaming motivation with different symptoms of IGD. For example, the associations between social gaming motivation and preoccupation with gaming activities and a deterioration in interpersonal relations were found to be stronger than the association between such motivation and academic problems [94]. Future studies should thus investigate the specific symptoms of IGD to allow comparisons of their potential differing associations with gaming motivation.

Finally, all the studies included in this systematic review adopted self-report questionnaires to assess both gaming motivation and IGD symptoms. The survey method is susceptible to various methodological biases in responding and recall [95], and no causal links can be inferred. Greater effort should be expended to the design of experiments or quasi-experiments to test the causal relationships between gaming motivation and IGD.

### Conclusions

This meta-analysis explores several issues in the study of gaming motivation and IGD. First, IGD was found to be more strongly associated with achievement motivation than with immersion motivation or social motivation; however, both of the latter components had weak positive associations with IGD. Second, our analysis at the subcomponent level strongly corroborates escape-from-self theory, with escapism motivation demonstrating a significantly stronger association than the other conceptually related subcomponents with IGD. Finally, our cross-cultural analysis identified the individualism-collectivism dimension to be a significant moderator of the association between escapism motivation and IGD, with a stronger such association found for studies conducted in individualistic countries than in those conducted in collectivistic countries.

## Abbreviations

DSM-5: Diagnostic and Statistical Manual of Mental Disorders
ICD-11: International Classification of Diseases, Eleventh Revision
IGD: internet gaming disorder
PRISMA: Preferred Reporting Items for Systematic Reviews and Meta-Analyses
